# Supplementary material for: Theoretical Study of Structure and Photophysics of Homologous Series of Bis(arylydene)cycloalkanones
Source: Int J Mol Sci. 2023 Aug 29;24(17):13362. doi: 10.3390/ijms241713362 (PMC10488151; doi:10.3390/ijms241713362)
Supplement: Supplementary file 1 [file ijms-24-13362-s001.zip › ijms-2543617-supplementary.pdf]

Supplementary materials

Theoretical Study of Structure and Photophysics of Homologous Series of Bis(arylydene)cycloalkanones

Roman O. Starostin, Alexandra Ya. Freidzon, Sergey P. Gromov

Table S1 Comparison of experimental [1–3] and calculated chemical shifts

			Cyclobutanones			
**Proton group**	4-H		4-OMe		3,4-OMe	
	Calculation	Experiment	Calculation	Experiment	Calculation	Experiment
C(3)H2	3.93	3.89	3.80	3.78	3.77	3.81
CH (methine)	7.36	7.19	7.23	7.11	7.20	7.10
			Cyclopentanones			
**Proton group**	4-H		4-OMe		3,4-OMe	
	Calculation	Experiment	Calculation	Experiment	Calculation	Experiment
C(3-4)H2	3.25	3.12	3.06	3.08	3.05	3.08
CH (methine)	7.86	7.6	7.67	7.56	7.66	7.51
			Cyclohexanones			
**Proton group**	4-H		4-OMe		3,4-OMe	
	Calculation	Experiment	Calculation	Experiment	Calculation	Experiment
C(3,5)H2	3.05	2.96	3.00	2.94	3.11	2.95
C(4)H2	1.69	1.80	1.70	1.80	1.75	1.81
CH (methine)	8.38	7.72	8.28	7.67	8.27	7.66

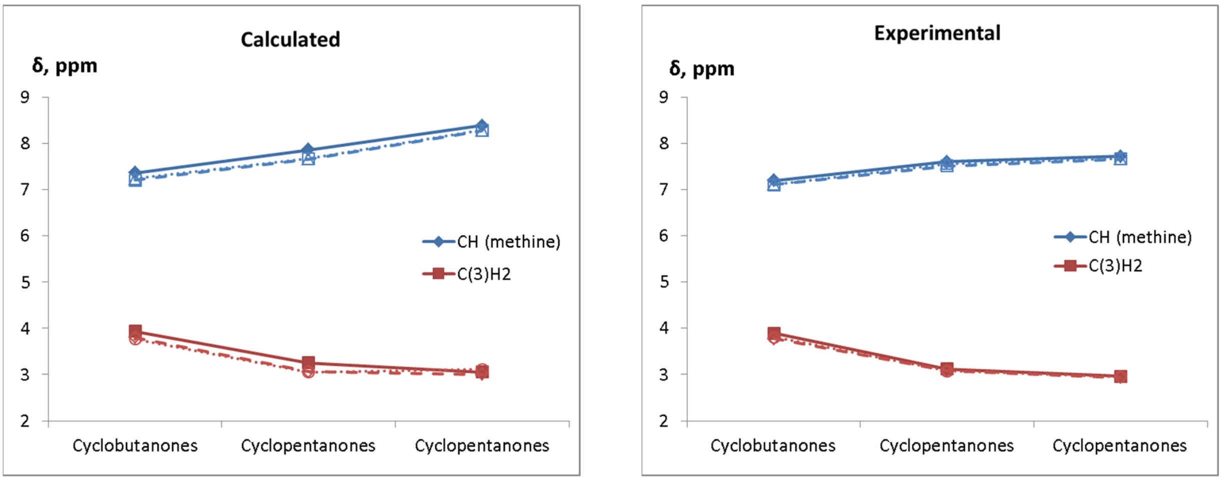

Figure S1 Calculated and experimental chemical shifts in 4-H (solid), 4-OMe (dashed), and 3,4-OMe (dotted).

Cyclobutanone

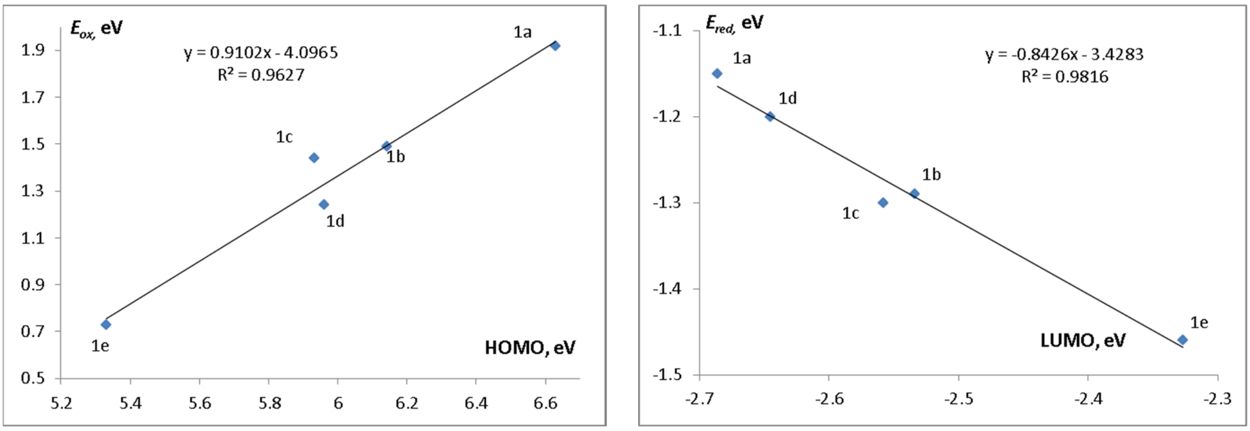

Cyclopentanone

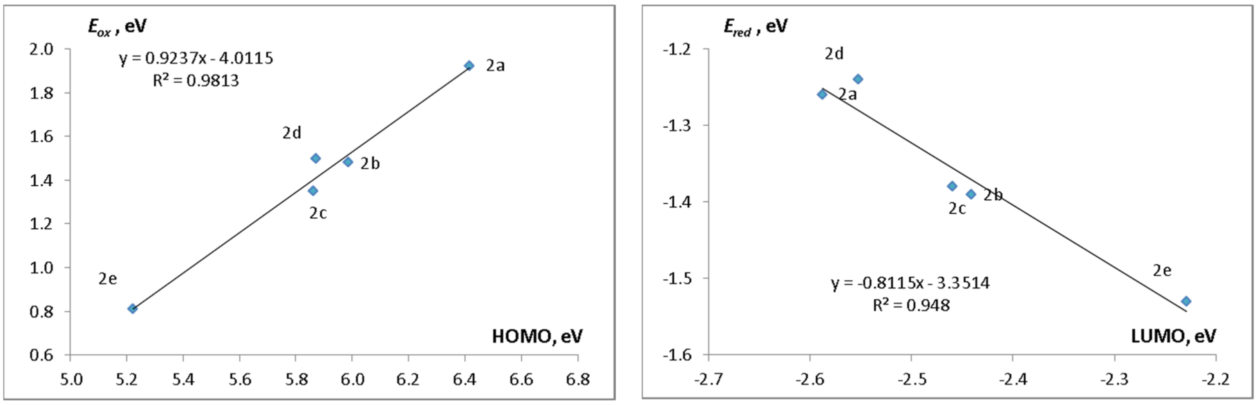

Cyclohexanone

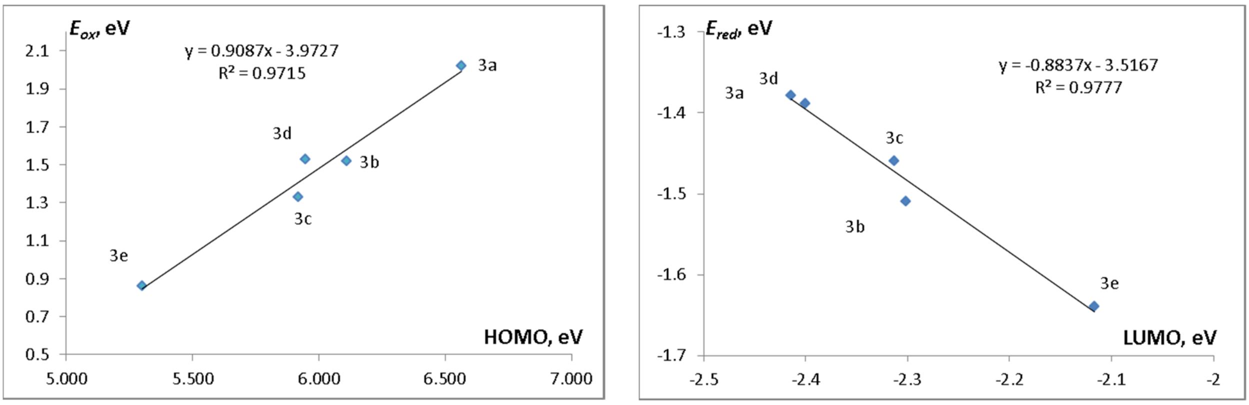

Figure S2 Correlations between the calculated HOMO and LUMO energies and experimental oxidation and reduction potentials [1–3].

Cyclobutanone

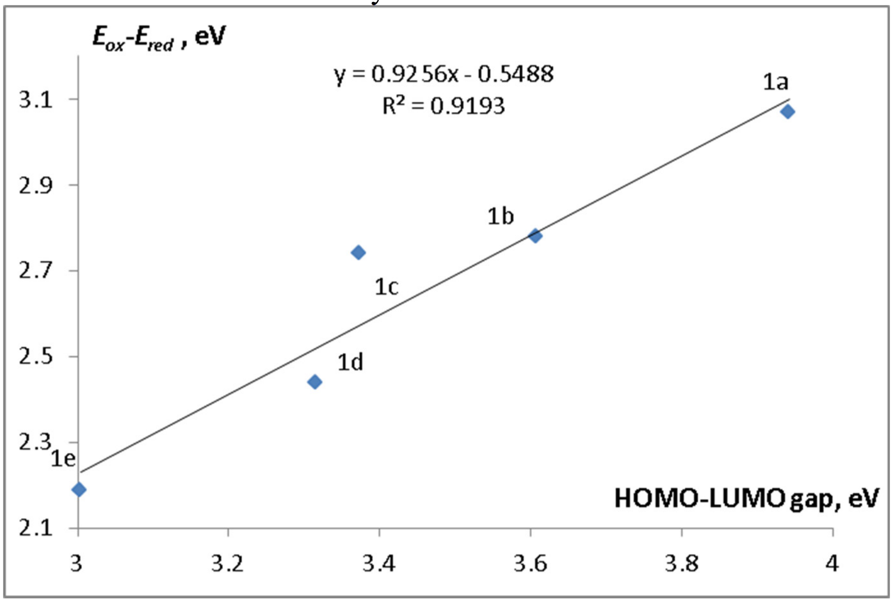

Cyclopentanone

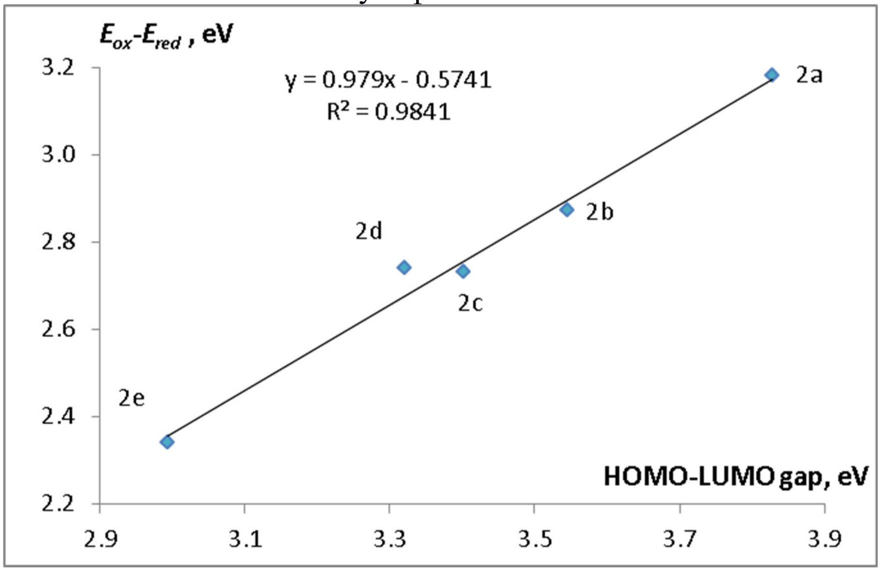

Cyclohexanone

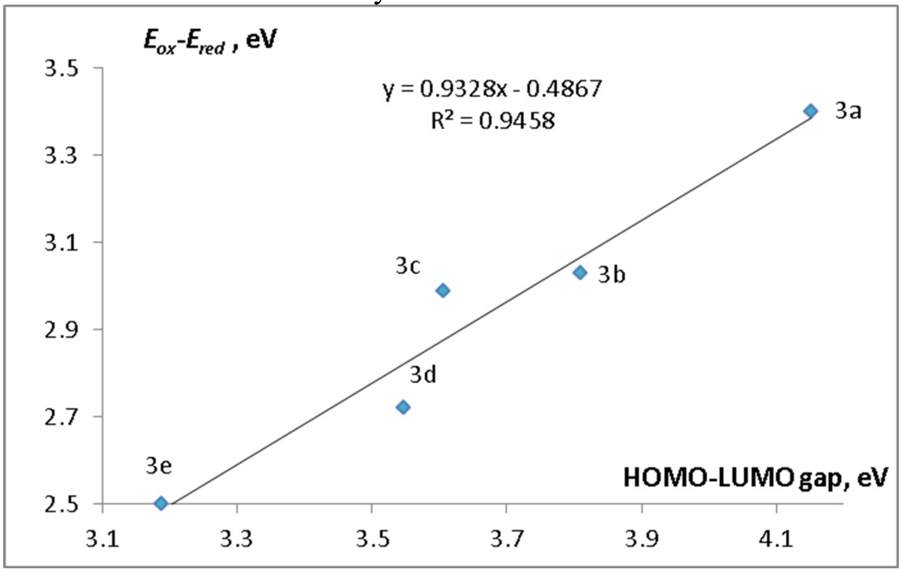

Figure S3. Correlations between the calculated HOMO–LUMO gap and experimental gap between the oxidation and reduction potentials [1–3].

Table S2. Calculated ionization potential, electron affinity, and IP–EA gap compared with the experimental oxidation and reduction potentials and E_{ox}–E_{red} gap [1–3]

	IP, eV	E_{ox}, V	EA, eV	E_{red}, V	IP–EA gap, eV	E_{ox}–E_{red}, V
			Cyclobutanones			
4-H	6.365	1.92	2.878	–1.15	3.487	3.07
4-OMe	5.873	1.49	2.716	–1.29	3.157	2.78
3,4-OMe	5.696	1.44	2.736	–1.3	2.960	2.74
4-SMe	5.746	1.24	2.827	–1.2	2.919	2.44
4-NEt2	5.073	0.73	2.481	–1.46	2.592	2.19
			Cyclopentanones			
4-H	6.147	1.92	2.795	–1.26	3.352	3.18
4-OMe	5.730	1.48	2.624	–1.39	3.107	2.87
3,4-OMe	5.622	1.35	2.630	–1.38	2.992	2.73
4-SMe	5.645	1.36	2.735	–1.38	2.910	2.74
4-NEt2	4.990	0.81	2.373	–1.53	2.617	2.34
			Cyclohexanones			
4-H	6.291	2.02	2.642	–1.38	3.650	3.4
4-OMe	5.840	1.52	2.513	–1.51	3.327	3.03
3,4-OMe	5.695	1.53	2.524	–1.46	3.172	2.79
4-SMe	5.724	1.33	2.618	–1.39	3.107	2.92
4-NEt2	5.059	0.86	2.304	–1.64	2.755	2.5

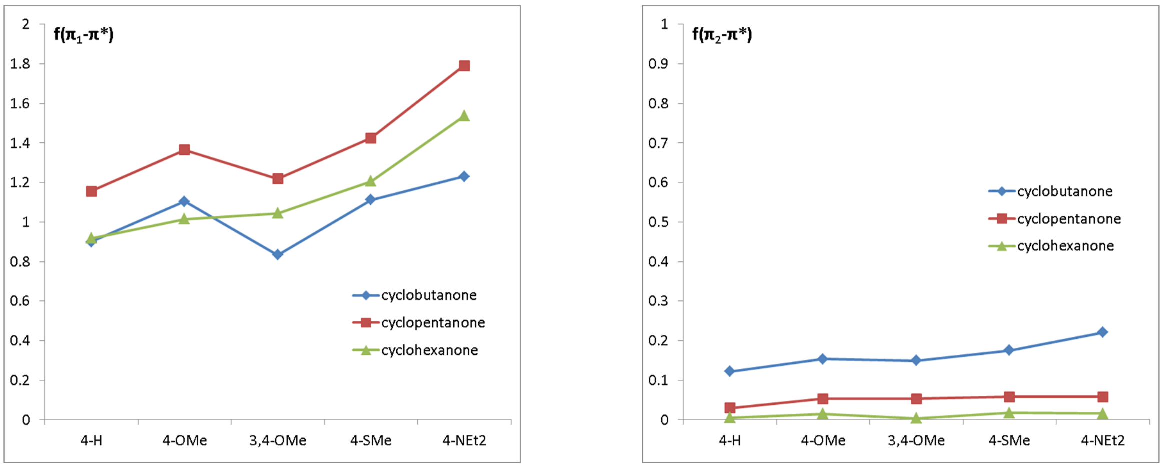

a **b**

Figure S4. Oscillator strengths of the first and second $\pi\pi^*$ transitions.

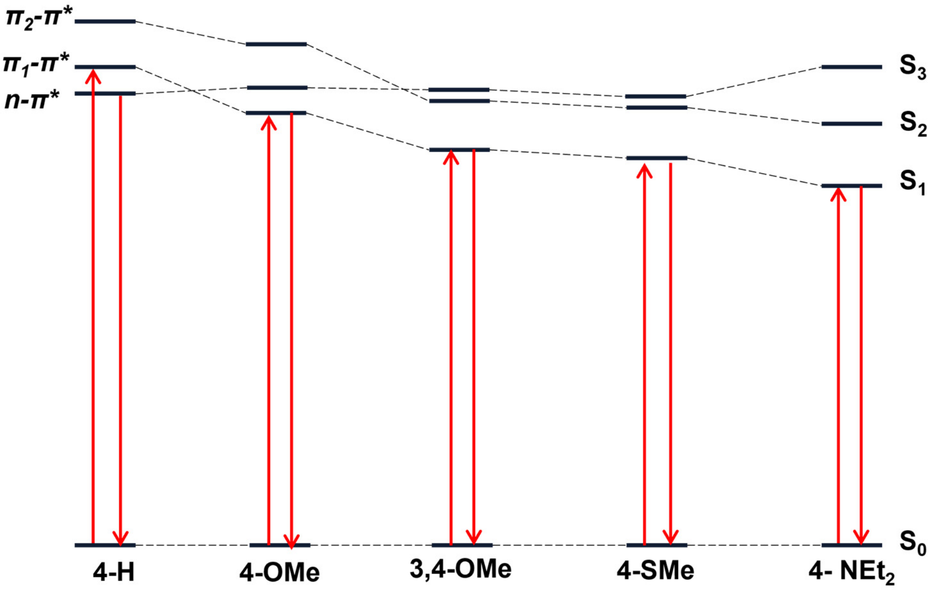

(a)

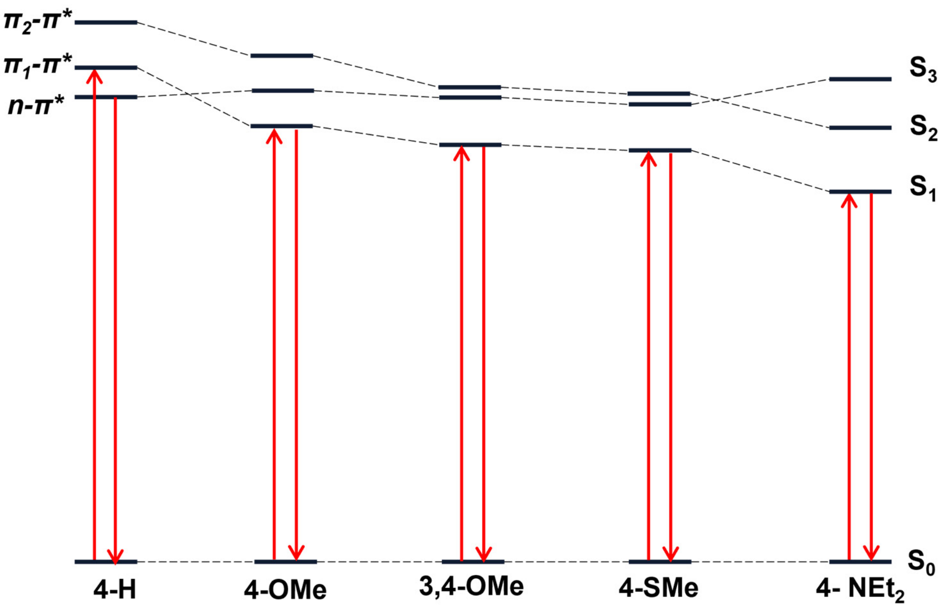

(b)

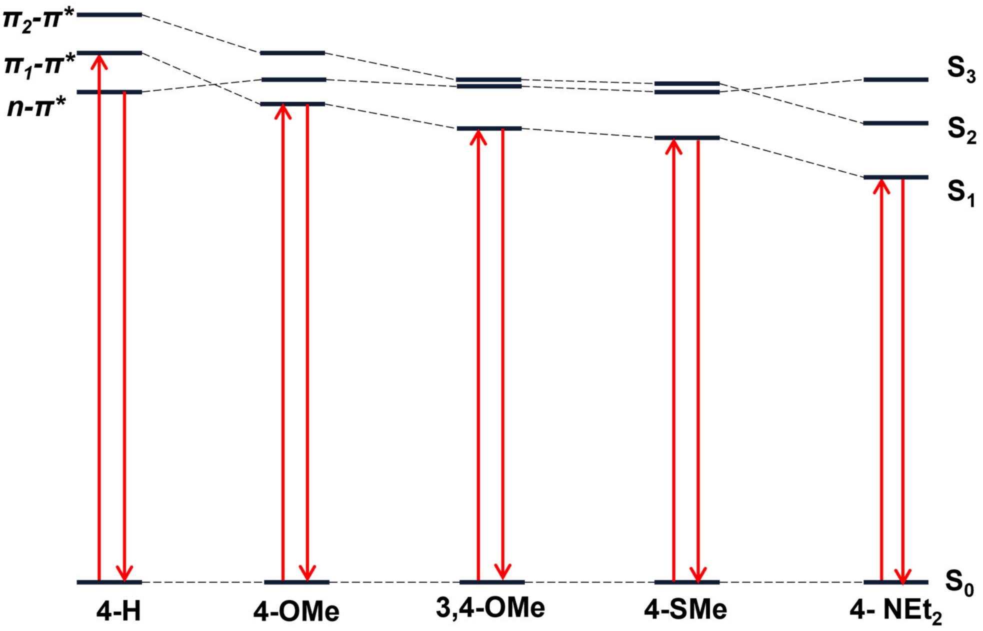

(c)

Figure S5. Energy diagrams of the excited states of (E,E) isomer in cyclobutanone (a), cyclopentanone (b) and cyclopentanone (c) series.

Table S3 Experimental [1–3] and calculated electronic transitions

Cyclobutanones						
		**λ_{abs}, nm**			**λ_{fl}, nm**	
**Dienone**	**Experiment**	**Calculation (Osc. Strength)**	**type**	**Experiment**	**Calculation (τ_{rad}, ns)**	**type**
**1a**	356	359 (0.90)<br>378 (2×10^{-7})	π–π*<br>n–π*	–	411 (2.3×10^{7})	n–π*
**1b**	385	393 (1.10)	π–π*	–	419 (2.28)	π–π*
**1c**	401	427 (0.83)	π–π*	505	446 (3.48)	π–π*
**1d**	402	432 (1.11)	π–π*	522	452 (2.62)	π–π*
**1e**	481	471 (1.23)	π–π*	575	491 (2.86)	π–π*
Cyclopentanones						
		**λ_{abs}, nm**			**λ_{fl}, nm**	
**Dienone**	**Experiment**	**Calculation (Osc. Strength)**	**type**	**Experiment**	**Calculation (τ_{rad}, ns)**	**type**
**2a**	347	371 (1.16)<br>379 (6.5×10^{-5})	π–π*<br>n–π*	–	401 (1.0×10^{7})	n–π*
**2b**	380	395 (1.36)	π–π*	–	422 (1.8)	π–π*
**2c**	395	420 (1.22)	π–π*	500	439 (2.2)	π–π*
**2d**	396	424 (1.42)	π–π*	508	458 (2.0)	π–π*
**2e**	471	472 (1.79)	π–π*	562	482 (1.9)	π–π*
Cyclohexanones						
		**λ_{abs}, nm**			**λ_{fl}, nm**	
**Dienone**	**Experiment**	**Calculation (Osc. Strength)**	**type**	**Experiment**	**Calculation (τ_{rad}, ns)**	**type**
**3a**	326	348 (0.92)<br>375 (0.07)	π–π*<br>n–π*	–	414 (778)	n–π*
**3b**	356	385 (1.02)	π–π*	–	431 (2.90)	π–π*
**3c**	370	404 (1.04)	π–π*	–	432 (2.26)	π–π*
**3d**	368	410 (1.21)	π–π*	–	441 (2.02)	π–π*
**3e**	445	455 (1.54)	π–π*	562	495 (2.46)	π–π*
